# Increased complexity of *Tmem16a*/*Anoctamin *1 transcript alternative splicing

**DOI:** 10.1186/1471-2199-12-35

**Published:** 2011-08-08

**Authors:** Kate E O'Driscoll, Rachel A Pipe, Fiona C Britton

**Affiliations:** 1 Department of Physiology and Cell Biology, 1664 North Virginia Street, University of Nevada School of Medicine, Reno, Nevada 89557-0046, USA

## Abstract

**Background:**

TMEM16A (Anoctamin 1; ANO1) is an eight transmembrane protein that functions as a calcium-activated chloride channel. *TMEM16A *in human exhibits alternatively spliced exons (6b, 13 and 15), which confer important roles in the regulation of channel function. Mouse *Tmem16a *is reported to consist of 25 exons that code for a 956 amino acid protein. In this study our aim was to provide details of mouse *Tmem16a *genomic structure and to investigate if *Tmem16a *transcript undergoes alternative splicing to generate channel diversity.

**Results:**

We identified *Tmem16a *transcript variants consisting of alternative exons 6b, 10, 13, 14, 15 and 18. Our findings indicate that many of these exons are expressed in various combinations and that these splicing events are mostly conserved between mouse and human. In addition, we confirmed the expression of these exon variants in other mouse tissues. Additional splicing events were identified including a novel conserved exon 13b, tandem splice sites of exon 1 and 21 and two intron retention events.

**Conclusion:**

Our results suggest that *Tmem16a *gene is significantly more complex than previously described. The complexity is especially evident in the region spanning exons 6 through 16 where a number of the alternative splicing events are thought to affect calcium sensitivity, voltage dependence and the kinetics of activation and deactivation of this calcium-activated chloride channel. The identification of multiple *Tmem16a *splice variants suggests that alternative splicing is an exquisite mechanism that operates to diversify TMEM16A channel function in both physiological and pathophysiological conditions.

## Background

Alternative splicing of pre-mRNAs is a powerful regulatory mechanism that can increase mRNA transcript variety and effect functional diversification of proteins [1]. Within the cardiovascular system, alternative splicing affects cardiac function by regulating proteins involved in cellular excitation, including ion channels [2-8].

Calcium-activated chloride currents have been recorded in cardiac muscle cells from various species including mouse [9], and play an important role in the cardiac action potential [10-12]. In 2008, three independent groups identified *Tmem16a *as a strong candidate gene to encode (or at least a major component of) a calcium-activated chloride channel [13-15]. *Tmem16a *belongs to a family of ten mammalian paralogs (*Tmem16 *(a-h, j-k)) that are highly conserved membrane spanning proteins. In recombinant expression systems, *Tmem16a *(or Ano1) and *Tmem16b *(or Ano2) generate calcium-activated chloride currents [13-18] with similar biophysical and pharmacological properties to currents recorded from native tissues [19]. We and others have identified *Tmem16a *expression in mouse and human heart [20,21].

The human *TMEM16A *gene exhibits three alternatively spliced exons (6b, 13 and 15) as well as an alternative transcription start site [13]. Ferrera et al. reported that the biophysical properties of human TMEM16A are regulated by alternative splicing and *TMEM16A *splice variants form functional channels that display different properties [22]. The alternative exon 6b (encoding 22 amino acids) may play an important role in the regulation of the TMEM16A channel by calcium, since exclusion of this exon increases the calcium sensitivity of the channel ~ 4-fold [22]. Exon 13, encoding 4 amino acids, contributes significantly to TMEM16A channel kinetics, since exclusion of this exon significantly reduces the voltage dependence of activation [22]. A recent study showed that exon 15 (encoding 26 amino acids) exclusion results in significantly faster activation and deactivation kinetics [23]. In addition, Mazzone et al, showed significant differences in expression of alternatively spliced *TMEM16A *exons in patients with diabetic gastroparesis when compared to non-diabetic controls [23]. Therefore, it appears that alternative splicing of human *TMEM16A *plays an important role in the regulation of calcium-activated chloride channel function.

The reported mouse *Tmem16a *gene [GenBank: NC_ 000073] is composed of 25 exons that code for a 956 amino acid protein [GenBank: NP_848757]. Unlike human, mouse *Tmem16a*, as annotated, does not contain alternative exons 6b, 13 or 15. We and others however, have reported that *Tmem16a *transcripts containing these alternative exons are expressed in mouse stomach, intestine [24] and vascular [25] smooth muscle tissues. It is likely that alternative splicing of *Tmem16a *transcript in mouse may lead to a number of different TMEM16A channel proteins with altered biophysical properties similar to human TMEM16A [22,23]. In this study our aim was to provide detailed information of the structure of the mouse *Tmem16a *gene and to investigate if *Tmem16a *transcript undergoes alternative splicing to generate channel diversity in mouse heart. This study demonstrates that the structure of *Tmem16a *gene is significantly more complex than previously indicated. The complexity is especially evident in the region containing exons 6 through 16. Determining the variation of *Tmem16a *transcript expression in heart is an important foundation for future studies of the physiological role of *Tmem16a *channels in heart.

## Methods

### RNA isolation and RT-PCR

Total RNA was isolated from mouse tissues using TRIzol reagent (Invitrogen, Carlsbad, CA). Human heart RNA was purchased from Agilent Technologies (Santa Clara, CA). First-strand cDNA was prepared from 1 μg of RNA using oligo(dT)_(12-18) _primer and Superscript II reverse transcriptase (Invitrogen). AmpliTaq Gold^® ^PCR reagent (Applied Biosystems, Foster City, CA) was used to amplify each of the *Tmem16 *paralogs and *Tmem16a *splice variants. PCR primers were designed using the mouse and human *Tmem16a *mRNA sequences [GenBank: NM_178642 and GenBank: NM_018043]. Details of the primer sets used are provided in additional file 1, Table S1 and Table S2). Glyceraldehyde-3-phosphate dehydrogenase (GAPDH) was used as a control for cDNA integrity. No template PCR reactions served as controls for primer contamination. PCRs were performed in a 2720 Thermal Cycler (Applied Biosystems). Amplification consisted of 95°C for 10 min, then 35 cycles of 95°C for 15 sec, T_a _for 20 sec and 72°C for 30-60 sec, followed by a final step at 72°C for 7 min.

### Splice variant identification, sequencing and bioinformatics

PCR products were resolved on 2-3% super fine agarose (Amresco, Solon, OH) gels along with a 100 bp molecular weight marker. *Tmem16a *amplification products were either purified (QIAquick Gel Extraction Kit, Qiagen, Valencia, CA) or TA cloned into the pcDNA3.1 vector (Invitrogen). All fragment sequencing was performed at the Nevada Genomics Center. Nucleotide and protein sequences were analyzed using Vector NTI version 11 software (Invitrogen) and BLASTN software at the National Center for Biotechnology Information (NCBI) database http://www.ncbi.nlm.nih.gov/BLAST/.

Transmembrane regions were predicted using the CBS TMHMM Server v 2.0 http://www.cbs.dtu.dk/services/TMHMM/ and TMpred http://www.ch.embnet.org/software/TMPRED_form.html programs. The TAndem Splice Site DataBase (TassDB2) http://gen100.imb-jena.de/TassDB2/ was used to confirm alternative tandem splice sites in mouse and human *Tmem16a*. The Scansite database http://scansite.mit.edu/motifscan_id.phtml was used to identify motifs in the TMEM16A alternative splice variants.

## Results and Discussion

### *Tmem16a *genomic structure is highly conserved in mouse and human

Genomic sequences for human [GenBank: NC_000011) and mouse [GenBank: NC_000073] *Tmem16a *were compared with their respective RNA sequence [GenBank: NM_018043 and NM_178642]. The reported mouse *Tmem16a *gene is composed of 25 exons, however, our analysis has revealed that mouse *Tmem16a *gene consists of at least 29 exons, ranging in size from 12 bp to 1621 bp. Table 1 provides details of mouse *Tmem16a *gene structure, including the number of exons, exon and intron size, 5' donor and 3'acceptor splice site sequences. The exon sizes of human *TMEM16A *are also provided for comparison purposes. The exon-intron junctions of *Tmem16a *comply with the consensus dinucleotide GT-AG rule. The number and size of each exon in mouse *Tmem16a *gene are highly conserved with human *TMEM16A*. There is some divergence between mouse and human *Tmem16a *genes at the 5' and 3' termini. For example, in mouse, exon 1 and exon 1b differ from human. The putative translational initiation codon of mouse and human *Tmem16a *are located in exon 1b and exon 1 respectively. The nucleotides adjacent to the initiator codons (mouse; GCCACCATGA and human; GCCACGATGA), constitute a favorable Kozak consensus initiation site [26]. Human *TMEM16A *has been reported to exhibit alternative splicing [13]. This study indicates that *Tmem16a *in mouse also exhibits multiple alternative exons, which generates numerous isoforms through alternative splicing of the primary transcript. A close examination of the mouse *Tmem16a *genomic sequence revealed that similar sequences to these human exon variants (exons 6b, 13 and 15) could be identified (Table 1). Additionally, a number of novel splicing events were identified (Table 1) and the details of these transcript variants are outlined throughout this report.

**Table 1:** Genomic Organization of *Tmem16a*

Human		Mouse			
**Exon ** **number**	**Exon size (bp)**	**Exon** **number**	**Exon size (bp)**	**3' Acceptor and 5' Donor splice site sequences**	**Intron** **size (bp) **

1	413	1	119	CAGGTG **gt**agga	~59,580
		1b	146	ttgc**ag **ACGCCA---------------------GGCACG **gt**gagt	8,958
2	333	2	333	tcac**ag **TTGCTG---------------------GAGGAT **gt**gagt	12,229
3	99	3	99	ctct**ag **ACCAAA---------------------AAGAAG **gt**gagt	1,264
4	152	4	152	ccac**ag **GTGTAC----------------------ACACCT **gt**gagt	1,340
5	55	5	55	ctgc**ag **ATTCGA----------------------ACAATA **gt**gagt	3,649
6	52	6	52	ccct**ag **GTCTAT---------------------GCATGG **gt**aagg	1,862
**6b**	66	**6b**	66	aaac**ag **GGCAAG--------------------AATACG **gt**aaga	495
7	56	7	56	ttaa**ag **GTATCA----------------------CACGAT **gt**aagt	3,274
8	42	8	42	tcac**ag **GGGGAC--------------------AGGAAA **gt**aagt	6,143
9	65	9	65	cccc**ag **CTCCTG----------------------GGTCAG **gt**aagt	1,290
**10**	135	**10**	135	gcat**ag **GAAATA---------------------CCCCAG **gt**aggc	3,318
11	161	11	161	ttac**ag **TATGGA----------------------TCTGGG **gt**aagt	11,845
12	83	12	83	ctac**ag **CTGCCA---------------------GAGGAG **gt**gagt	2,181
**13**	12	**13**	12	ccgc**ag **GAAGCT---------------------GTCAAG **gt**ttga	161
		**13b**	120	ttcc**ag **CACCTT----------------------CAGAGG **gt**cagt	273
**14**	72	**14**	72	tctc**ag **GATCAT---------------------AAAGAG **gt**acag	2,874
**15**	78	**15**	78	cctc**ag **AAGTGC----------------------AAATTG **gt**actt	972
16	75	16	75	ttgc**ag **ACCGAC----------------------TTCATG **gt**aagt	3,034
17	202	17	202	ctgc**ag **ATCGCA----------------------AGATTG **gt**gagt	167
**18**	112	**18**	112	cctc**ag **AGGTCC---------------------AGGCCG **gt**agga	434
19	58	19	58	tccc**ag **GTTTGT---------------------GAGGAG **gt**aata	1,892
20	101	20	101	ctac**ag **TGTGCC----------------------CATCCC **gt**gagt	786
21	146	21	146	ctgc**ag **GAAGAT---------------------AAATGA **gt**gagt	4,324
22	153	22	153	ctgc**ag **TCATTC-----------------------ACATCG **gt**aagt	6,207
23	53	23	53	ctct**ag **GCATCT-----------------------ATTAAT **gt**aagt	1,554
24	185	24	185	tggc**ag **GCCTTT----------------------CTGCAG **gt**acta	2,635
25	106	25	106	ttgc**ag **GTATAA-----------------------TTCCAG **gt**atgt	2,435
26	1809	26	1621	ctgc**ag **AACCTG	

Intron size refers to the intron following the exon listed in each row.

### Identification of *Tmem16a *splice variant expression in mouse heart

**Figure 1:**
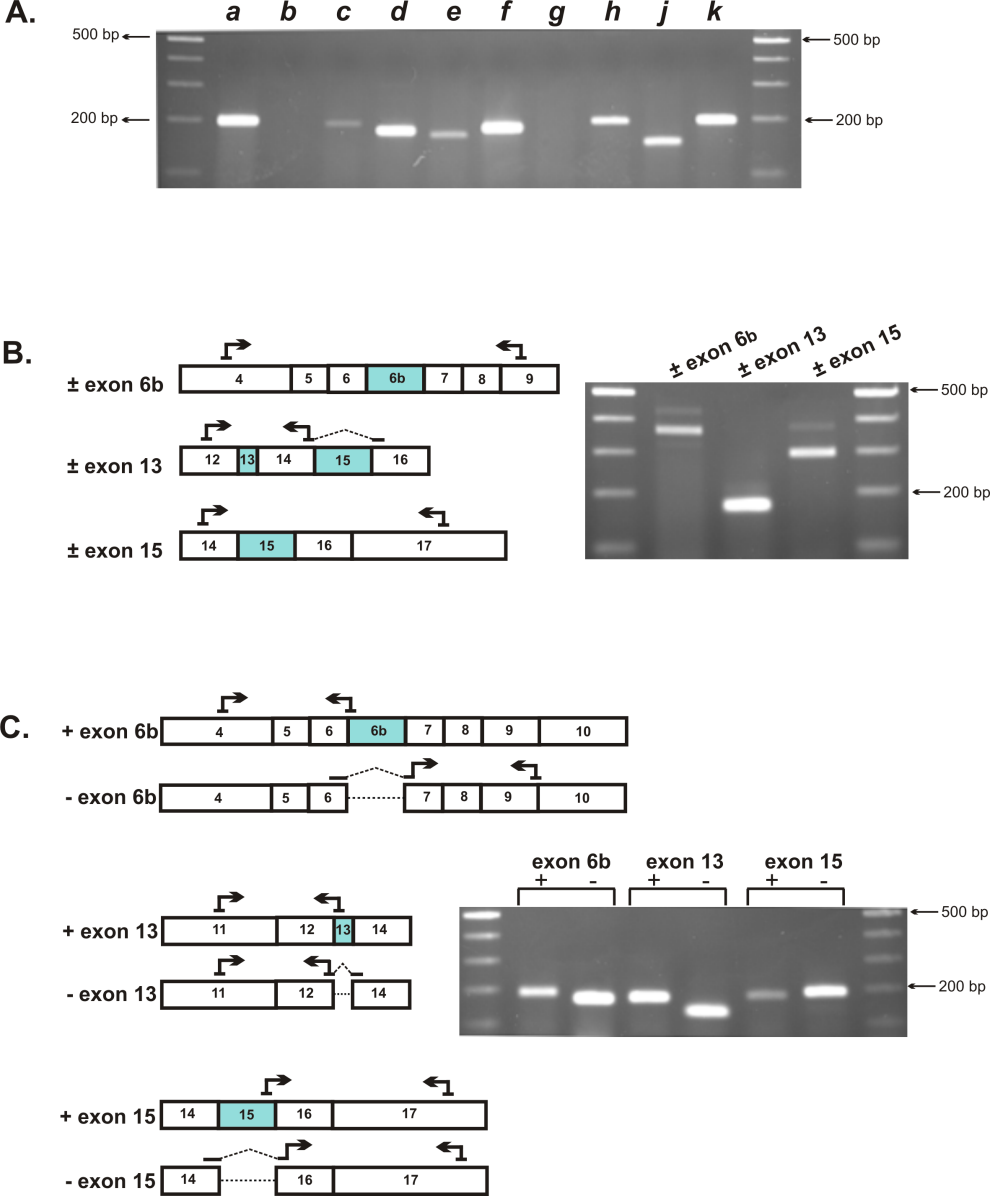
**Identification of alternative *Tmem16a *exons 6b, 13 and 15 in mouse heart**. **A**. Expression of *Tmem16 *family members were analyzed in mouse heart using RT-PCR primers specific for each of the ten *Tmem16 *paralogs. Amplification products were resolved on 2% agarose gels alongside a 100 bp marker. Fragments of the expected molecular size indicated the expression of *Tmem16a*, *c*, *d*, *e*, *f*, *h*, *j *and *k *paralogs in mouse heart. *Tmem16b *and *g *were not detected. **B**. Schematic representations of regions of *Tmem16a *RNA. Boxed numbers indicate exons relative to the *Tmem16a *gene. Alternative exon 6b (66 bp), exon 13 (12 bp) and exon 15 (78 bp) in the mouse *Tmem16a *coding sequence are highlighted. RT-PCR primers (± exon 6b, ± exon 13, ± exon 15), which amplify across each alternative exon are indicated. RT-PCR gel analysis of exon 6b, 13 and 15 *Tmem16a *variants expressed in mouse heart. Primers ± exon 6b amplify a 367 and/or a 433 bp product, primers ± exon 13 amplify a 165 and/or a 177 bp product, primers ± exon 15 amplify a 297 and/or a 375 bp product. The upper bands correspond to *Tmem16a *variants that include the alternative exon and differ in size corresponding to the size of that exon. **C**. RT-PCR primer sets that span exon boundaries to selectively amplify individual alternative exons are indicated. Gel analysis of *Tmem16a *fragments expressed in mouse heart that either include or exclude exons 6b, 13 or 15. Each primer set yielded a single band of the expected size. RT-PCR products were sequenced for confirmation.

RT-PCR was initially used to identify the expression of *Tmem16 *paralogs in mouse heart using primers specific for each of the *Tmem16 *family members (Additional file 1, Table S1). In addition to *Tmem16a*, several other *Tmem16 *transcripts were detected in mouse heart: *Tmem16c, d, e, f, h, j *and *k *(Figure 1A). The expression of *Tmem16b *and *Tmem16g *transcripts were not detected. To determine if alternative exons, homologous to the exons 6b, 13 and 15 of human *TMEM16A *[13], are also expressed in mouse heart we performed RT-PCR analysis of *Tmem16a *(Figure 1B). Primer sets ± exon 6b, ± exon 13 and ± exon 15 amplify fragments that differ in size (± 66 bp, 12 bp and 78 bp, respectively) corresponding to the size of the alternative exon. The amplification of two fragments using ± exon 6b and ± exon 15 primers clearly indicates that *Tmem16a *variants that either include or exclude these alternative exons are expressed in mouse heart (Figure 1B). The presence or absence of the alternative 12 bp micro-exon 13 is difficult to resolve by gel electrophoresis, therefore additional primers were designed that either hybridize within each of the alternative exons to specifically amplify them or that span bordering exons to exclude them (Figure 1C and Additional file 1, Table S2). For example, *Tmem16a *transcripts lacking exon 15 (-exon 15) were selectively amplified using a primer that spans the junction of exons 14 and 16. Similarly, *Tmem16a *transcripts containing exon 15 (+exon 15) were specifically amplified using a primer internal to exon 15. RT-PCR gel analysis (Figure 1C) and fragment sequencing confirmed that *Tmem16a *variants are expressed in mouse heart that either exclude or include exon 6b, exon 13 and exon 15, identical to those we identified in mouse gastrointestinal tissues [24].

A comparison of the amino acid sequences of mouse and human alternative exons show a high similarity; the four amino acids (EAVK) of exon 13 are 100% identical, the 22 and 26 amino acids encoded by exon 6b and exon 15, share 95.5% and 80.8% identity, respectively. Considering the high percentage amino acid identity of these exon between the two species, we can expect that the regulatory properties attributed to these channel domains in human [22,23], will most likely confer similar functional roles in the mouse, in that, exon 6b may confer calcium sensitivity, exon 13 may contribute to voltage dependence and exon 15 may play a role in the activation and deactivation kinetics of this calcium activated chloride channel. The inclusion of alternative exons 6b, 13 and 15 would result in additional amino acids in intracellular channel domains and are not predicted to alter the eight transmembrane topology of the TMEM16A protein.

### *Tmem16a *transcripts with combinations of alternative exons 6b, 13 and 15 create diversity

**Figure 2:**
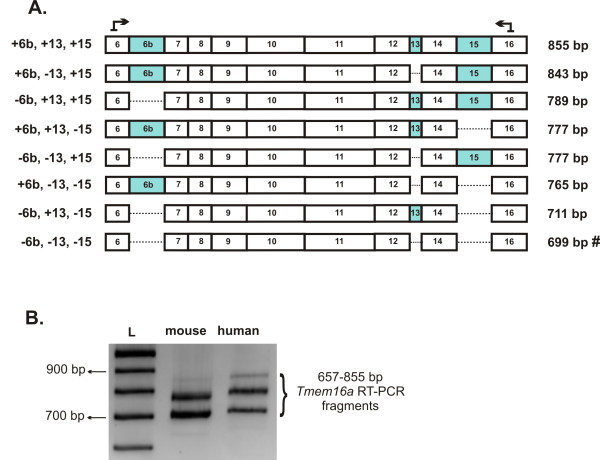
**Multiple *Tmem16a *variants are expressed in heart due to different combinations of alternative exons 6b, 13 and 15**. **A**. Cartoon indicating eight possible *Tmem16a *transcript variants as a result of combinations of exon 6b, 13 or 15 inclusion and/or exclusion. Dashed lines denote the exclusion of an alternative exon. Primers which span *Tmem16a *from exon 6 to exon 16, a region encompassing the three alternative exons (shaded), generate transcripts of various sizes depending upon which exons are amplified. The expected sizes of RT-PCR products are indicated. *Tmem16a *variant [GenBank: NM_178642] lacking all 3 exons is indicated (**#**). **B**. Gel analysis of *Tmem16a *RT-PCR fragments ranging between 657 and 855 bp were amplified. 100 bp ladder (L).

A detailed investigation into the expression of various alternative exon combinations has not been reported for *Tmem16a*. To explore if different combinations of these alternative exons exist in mouse and human heart, we employed RT-PCR analysis to examine the region of the *Tmem16a *transcript encompassing alternative exons 6b, 13 and 15 (Figure 2). Eight *Tmem16a *variants may be generated due to alternative combinations of exon inclusion or exclusion (Figure 2A). RT-PCR analysis generated *Tmem16a *transcripts of various molecular sizes expressed in mouse and human heart (Figure 2B), corresponding to different combinations of *Tmem16a *alternative exon 6b, 13 or 15 inclusion or exclusion. The complexity of alternative exon combinations is difficult to resolve by size analysis alone, largely due to the micro exon 13 and two possible fragments of the same size (Figure 2A). Thus, the entire RT-PCR amplifications were cloned, and sequence analysis of multiple clones confirmed that at least six distinct *Tmem16a *splice variants consisting of various combinations of exons 6b, 13 and 15 are expressed in mouse and human heart.

Both mouse and human heart express the following three exon combinations: *Tmem16a *(-6b, +13, -15), *Tmem16a *(+6b, +13, -15), and *Tmem16a *(-6b, +13, +15). We did not identify *Tmem16a *transcripts consisting of exon 6b alone (+6b, -13, -15) or a *Tmem16a *variant containing 6b and 15 but lacking exon 13 (+6b, -13, +15) in either mouse or human heart. It appears, at least in heart, that exon 6b is only expressed in the presence of exon 13. However, we did identify *Tmem16a *transcripts containing exon 15 in the absence of both exons 6b and 13 (-6b, -13, +15) in mouse heart. Interestingly, a *Tmem16a *transcript missing all three exons (-6b, -13, -15) was only identified in mouse heart, in agreement with the reported *Tmem16a *[GenBank: NM_178642] and although a human transcript variant 2 (-6b, -13, -15) [GenBank: NR_030691] exists we did not confirm its presence in human heart. All human *TMEM16A *transcripts contained exon 13, in agreement with [GenBank: NM_018043] (-6b, +13, +15). The largest *Tmem16a *variant consisting of all three exons was confirmed by sequence analysis in human heart. Although this *Tmem16a *variant was not confirmed in our screening of mouse heart clones, we cannot rule out its existence since our gel analysis indicates a band close to the expected size of this variant, albeit much weaker than in human (Figure 2B). Indeed, it is possible that different mouse and human tissues may express different combinations of these alternative exons, however, quantification of our data would not provide more information since the tissues examined in this study contain a mixed population of cells. The deduced amino acid sequence of mouse TMEM16A containing alternative exons combinations (6b, 13 and 15) all read in-frame, indicating that these alternative exons are additional channel components similar to those of human TMEM16A [22]. BLASTn searches of dbEST and Refseq RNA databases identified a number of mouse ESTs containing exon 6b and exon 13. Indeed, one mouse EST [GenBank: BI151426] was identified for the +6b, +13, -15 exon combination. EST supporting query sequences were identified for mouse [GenBank: BI685387] containing exon 13 alone (-6b, +13, -15). No ESTs were found in mouse containing both exon 13 and 15, however this is the exon combination in human that corresponds to *TMEM16A *[GenBank: NM_018043].

In addition to alternative exons 6b, 13 and 15, we also identified a number of novel *Tmem16a *splicing events in mouse and human heart; exon 10 and exon 14 exclusion, the inclusion of a novel exon, and two intron retention events.

### Novel *Tmem16a *transcripts with exon 10 or exon 14 exclusion

**Figure 3:**
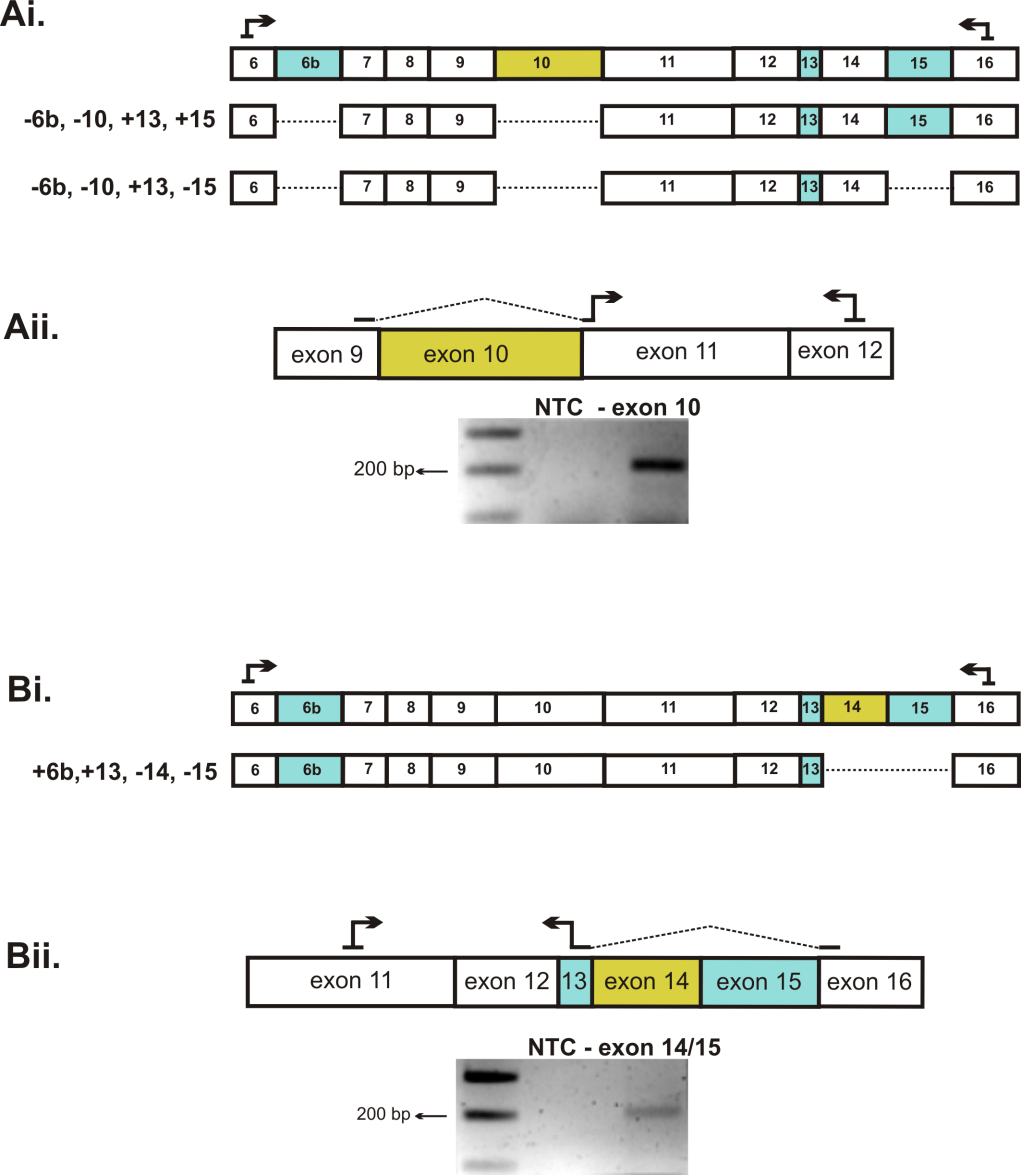
**Identification of *Tmem16a *novel exon 10 and exon 14 skipping events**. **Ai**. Cartoon indicating *Tmem16a *variants as a result of combinations of exon 10 exclusion, (-6b, -10, +13, +15) and (-6b, -10, +13, -15). Dashed lines denote the exclusion of an alternative exon. **Aii**. RT-PCR with a specific primer set to verify the exclusion of exon 10 in mouse heart **Bi**. Cartoon indicating the *Tmem16a *variants as a result of exon 14 exclusion, (+6b,+13, -14, -15). **Bii**. RT-PCR with a specific primer set to verify the exclusion of exon 14 in mouse heart.

We identified two previously unreported *Tmem16a *exon skipping events, exon 10 and exon 14, in mouse and human heart (Figure 3). Exclusion of exon 10 (135 bp) in *Tmem16a *RNA would result in an in-frame deletion of 45 amino acids in the channel protein. Exon 10 encodes a hydrophobic region predicted to be the first transmembrane spanning domain of TMEM16A. Therefore, exon 10 exclusion would result in a change in the transmembrane topology. We identified through sequencing two different *Tmem16a *(-10) transcripts, in that both transcripts lacked exon 6b and included exon 13, however, they differed in whether or not exon 15 was included. This is highlighted in the cartoon in Figure 3A. We employed an additional primer set to specifically amplify *Tmem16a *(-10) variants (Figure 3A) and sequence analysis confirmed the presence of these transcript variants in mouse and human heart.

Sequence analysis revealed that the *Tmem16a *(-14) variant included exons 6b and 13 and excluded exon 15 (Figure 3B). Exclusion of exon 14 (72 bp) in *Tmem16a *RNA would result in an in-frame deletion of 24 amino acids in the first intracellular loop of the TMEM16A channel. A PKC site is present in exon 14 at position S462 [GenBank: NP_848757] and one could speculate that *Tmem16a *transcripts lacking exon 14 may encode channels that differ in modulation by PKC. Likewise, the expression of this variant was confirmed in mouse and human heart using primers which specifically amplify *Tmem16a *(-14) variants.

### *Tmem16a *transcripts with a novel conserved exon 13b

**Figure 4:**
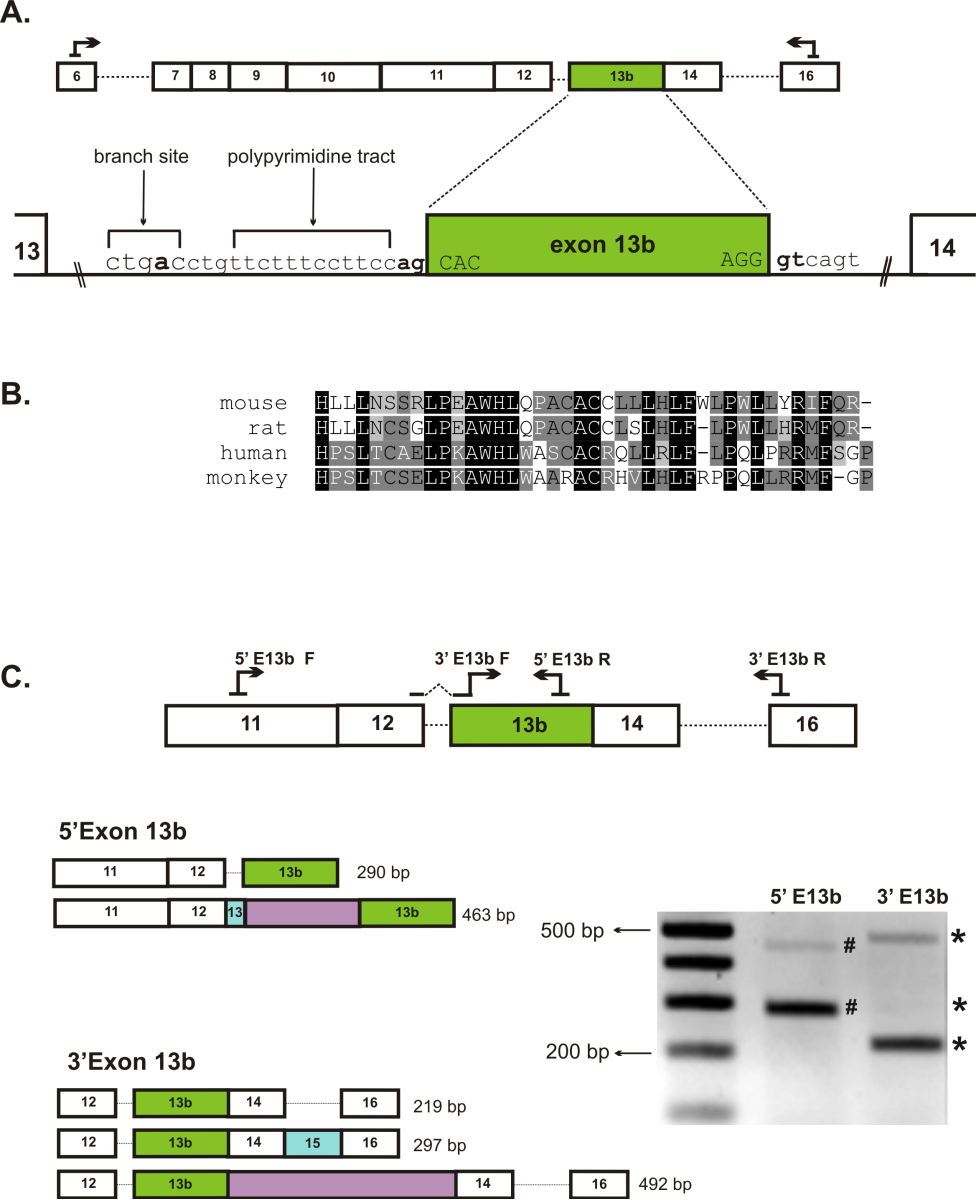
**Identification of a novel exon 13b in *Tmem16a***. **A**. Cartoon representation of the novel exon 13b identified through sequence analysis of a *Tmem16a *fragment amplified using primers that span exons 6 through 16. Exon13b (120 bp) is highlighted. The genomic region between exon 13 and 14 is expanded to indicate the location of exon 13b, the splice and branch sites and the polypyrimidine tract. **B**. Alignment of the 40 amino acids encoded by exon 13b of mouse with the corresponding residues identified in rat, human and rhesus monkey. **C**. RT-PCR with primer sets 5'Exon13b and 3'Exon13b verified the expression of exon 13b in mouse heart. Cartoon of the amplification products generated with these primers is shown. Primer set 5'Exon13b produced two fragments, the 290 bp fragment consisted of exons 11, 12 and 13b; the 463 bp fragment consisted of exons 11, 12, 13 and13b plus the entire intronic sequence between exon 13 and 13b. The 3'E13b primer set yielded three fragments. The 219 bp and 297 bp fragments included exons 12, 13b, 14, ± 15, 16, and the 492 bp fragment consisted of 12, 13b, 14, -15, 16 plus the intronic sequence between exon 13b and 14. The intron retention events are highlighted.

Sequence screening of our *Tmem16a *clone library, identified an unusual *Tmem16a *transcript that excluded alternative exons 6b, 13 and 15 yet contained an additional 120 nucleotides between exon 12 and exon 14. Evaluation of *Tmem16a *genomic sequence revealed that these 120 nucleotides are located within the 554 bp intronic region between exon 13 and 14 (Figure 4A) and are bordered by conserved 'GT-AG' acceptor and donor splice sites [27]. We assigned the additional sequence in this *Tmem16a *transcript as exon 13b. A close examination of the intronic sequence immediately upstream of exon 13b identified a conserved mammalian "CURAY" branchpoint sequence 18 nucleotides upstream of the 3' splice site (Figure 4A). The branchpoint (A nucleotide) is generally situated 18 to 40 nucleotides upstream of the 3' splice site and plays an important role in identifying this splice site [28,29]. An adjacent polypyrimidine tract, which increases the efficiency of branchpoint utilization [30], was also identified in this region (Figure 4A). Together, these findings add support that this 120 bp sequence is a true exon. Inclusion of exon 13b in the *Tmem16a *transcript would result in an in-frame addition of 40 amino acids in TMEM16A. This region is rich in hydrophobic leucine residues (12/40 residues being leucines). It is not surprising then that hydropathy analysis suggests that exon 13b inclusion may constitute an additional domain in the TMEM16A channel integral to the membrane.

Considering that the exon structures of *Tmem16a *are highly conserved amongst homologs, we investigated if similar novel exons could be identified in other species. A close examination of the genomic *Tmem16a *sequence from rat, human and rhesus monkey [GenBank: NC_005100, GenBank: NC_000011, GenBank: NC_007871,] revealed that similar sequences to mouse *Tmem16a *exon 13b, bordered by conserved splice sites could be identified. An alignment of the corresponding amino acid sequence of these four species (Figure 4B) demonstrates 97.6% overall consensus with an identity of 41.5%. Specific primers designed to amplify this novel exon in human heart were unable to identify its expression. Whether or not this exon is present in other human tissue types is unknown.

The expression of the novel exon 13b in mouse heart was investigated further using primer sets to specifically amplify the 5' and 3' regions surrounding the 120 bp exon (Figure 4C). Sequence analysis revealed some interesting observations. First, we confirmed that exon 13b is utilized in mouse heart. Secondly, exon 13b is present in several *Tmem16a *transcripts that differ in whether they also include or exclude alternative exons 13 or 15. Third, retention of the introns surrounding exon 13b increases the complexity of splicing in this region of *Tmem16a *transcript. RT-PCR with the primer set 5'Exon13b, in which primers bind within exon 11 and 13b produced two amplification products (Figure 4C). Sequencing analysis indicated one *Tmem16a *fragment consisted of exons 11, 12 and 13b and the other fragment consisted of exons 11, 12, 13, 13b plus the entire 161 bp intronic sequence between exon 13 and 13b (Figure 4C). The 3'Exon13b primer set, in which the primers hybridize across exon 12/13b junction and within exon 16, yielded three *Tmem16a *transcript fragments (Figure 4C). Two fragments differ in exon 15 inclusion/exclusion exons 12, 13b, 14, ± 15, 16, and an unexpected fragment consisting of exon 12, 13b, 14, -15, 16 plus the entire intronic sequence (273 bp) between exon 13b and 14 (Figure 4C). Intron retention events at the 5' and 3' of exon 13b are certainly the result of alternative *Tmem16a *splicing and not genomic contamination since the 5'Exon13b and 3'Exon13b primers span at least 14.4 kb and 4.4 kb of genomic sequence, respectively.

The intron retention events at the 5'and 3' of exon 13b would introduce a premature stop codon in the *Tmem16a *transcript, resulting in a truncated protein just after the "EAVK" amino acids encoded by exon 13. The complete retention of an intron in a mature transcript is one of the least expected forms of alternative splicing and may occur due to weak splice sites, cis-regulatory elements or short intron lengths [31]. Indeed, the introns between exons 13 and 13b and between exon 13b and 14 are two of the shortest in the *Tmem16a *genomic sequence (see Table 1). Intron retention can result in the insertion of a premature stop codon being introduced to the mature transcript which may then be degraded by nonsense mediated decay [32]. Indeed, decreasing the mRNA levels of certain genes can be the purpose of alternative splicing [32]. However, sometimes the retention of an intron in the coding region can have a biological effect. For example, an intron retaining isoform of the *KCNMA1 *gene, which encodes the large conductance calcium-activated potassium channel (BK), facilitates splice variant regulation which contributes to structural and functional diversity of BK channel proteins in hippocampal neurons [33,34].

### *Tmem16a *transcript scanning assay reveals additional novel splice variants

It is clear from our analysis that *Tmem16a *transcript undergoes extensive splicing within the region between exons 6 and 16. In addition, not all of these exon inclusion/exclusion combinations are consistent between mouse and human. Indeed splicing events occurring at other regions of *Tmem16a *transcript may have important functional implications. Thus, we profiled the entire *Tmem16a *using overlapping RT-PCR primers to determine whether additional *Tmem16a *RNA splicing events occur in mouse and human heart. The transcript scanning assay and sequencing analysis revealed additional novel *Tmem16a *variants. These variants included exon 18 exclusion in both mouse and human *Tmem16a *and two alternative 3' donor sites, Δ3'exon 1 and Δ3'exon 21, in mouse. Details for mouse and human *Tmem16a *are summarized in Tables 2 and 3.

**Table 2:** Mouse *Tmem16a *transcript scanning assay

Primer Set	Oligonucleotid sequences (5'→ 3')	*Tmem16a * region	Size (bp)	Alternative exon(s) identified
A	GAGGGAACCTCTGCGGACCGA (sense)	Exons 1-3	613	
	GCCAGGGCGCATGGATCTTC (antisense)		**610**	Δ3' Exon 1
B	GGCCTGGAGTTGGAGAATGACG (sense)	Exons 2-10	602	
	GTGTAGGCTCCAAGCCAGGCAA (antisense)		**668**	+ Exon 6b
C	GAACAACGTGCACCAAGGCCAAGTA (sense)	Exons 6-16	699	
	TGGTGAAATAGGCTGGGAATCGGTC (antisense)		**711**	+ Exon 13
			**777**	+ Exon 15
			**777**	+ Exons 6b + 13
			**789**	+ Exons 13 + 15
			**819**	+ Exon 13b
			**654**	- Exons 10 +13 +15
			**705**	- Exons 6b +13 -14
D	TGGAGCACTGGAAACGGAAG (sense)	Exons 12-20	625	
	ATACAGAGCTCCATGAGGCAGC (antisense)		**637**	+ Exon 13
			**715**	Exons 13 + 15
E	ACCGACAAGGTGAAGCTGAC (sense)	Exons 16-22	731	
	GACGCAACAAACAGGGTGAC (antisense)		**619**	- Exon 18
			**623**	Δ3' Exon 21
F	GAAGTTTACGGCTGCATTGC (sense)	Exons 17-24	719	
	ATGTAGAGGTACACCAGGCGAG (antisense)			
G	GCCTCATGGAGCTCTGTATCC (sense)	Exons 20-26	767	
	CACCCAGTCCACAAAGTCACTC (antisense)			
H	TGGTATAACATCCTCAGAGGTG (sense)	Exons 23-26	656	
	TCACTTGGTGATGGTCAGAA (antisense)			

**Table 3:** Human *TMEM16A *transcript scanning assay

Primer Set	Oligonucleotide sequences (5'→ 3')	*Tmem16a * region	Size (bp)	Alternative exon(s) identified
A	ACAGGCGGCCACGATGAGGGTC (sense) GAGCCTGCATCCAGCGACGCC (antisense)	Exons 1-2	348	
B	AGGAGGGTGCAGCACAGCGA (sense) ATTGGCCAGCAGGCTCGTGA (antisense)	Exons 2-7	582 **648**	+ Exon 6b
C	GAGAACGACGTGTACAAAGGCCAAG (sense)	Exons 6-16	**714**	+ Exon 13
	TTAGTGAGGTAGGCTGGGAACCGAT (antisense)		**780**	+ Exons 6b + 13
			792	+ Exons 13 + 15
			**858**	+ Exons 6b +13 **+**15
			**579**	- Exons 10 + 13
			**657**	- Exons 10 + 13+15
			**708**	+ Exons 6b + 13 - 14
D	ACTGACAAAGTGAAGCTGACA (sense)	Exons 16-20	526	
	TGTTCTGGATCAGCTGTTTC (antisense)		**414**	- Exon 18
E	TTTCCGTTCCTTCCGAATGG (sense)	Exons 19-23	460	
	CTTCCCAATGCCTCTGAGGATA (antisense)			
F	TGTCACTGAGCTCCGAAGGCCG (sense)	Exons 22-25	384	
	ACGATGACAAACGCCAGCCGG (antisense)			
G	AGTAAGAACGGGACCATGCACGG (sense)	Exons 24-26	509	
	CGCTGGCATAGCTACAGGACGC (antisense)			

**Figure 5:**
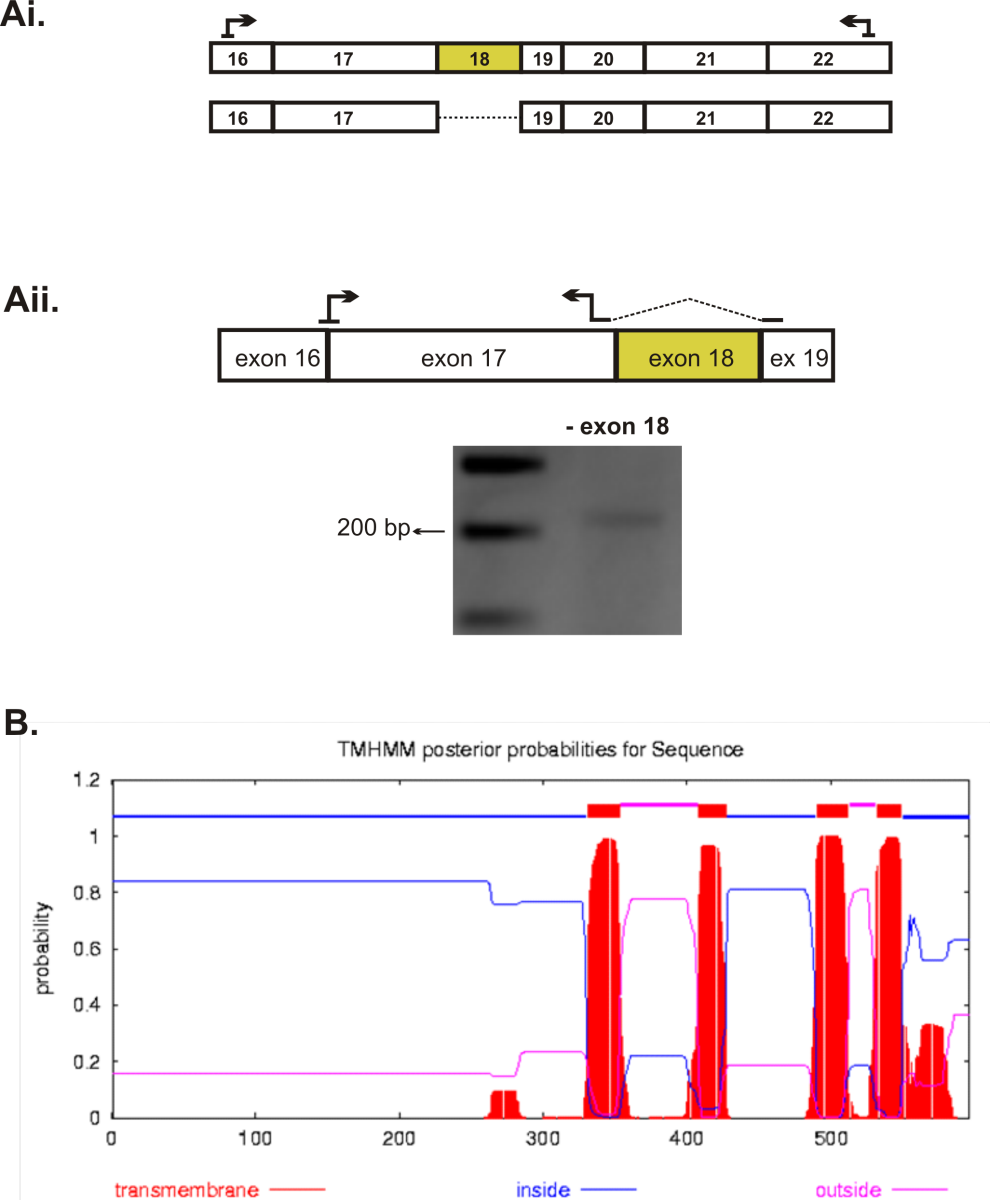
**Exon 18 deletion truncates the TMEM16A protein**. **A**. Cartoon indicating the *Tmem16a *variant as a result of exon 18 exclusion identified in the transcript scanning assay. RT-PCR with a specific primer set (spanning the boundary of exons 17 and 19) verified the exclusion of exon 18 in mouse heart. **B**. Exon 18 exclusion truncates TMEM16A protein to 596 amino acids and the predicted hydropathy profile is shown. Red boxes represent the four transmembrane spanning domains, blue and pink lines represent intracellular and extracellular locations respectively.

Exon 18 is 112 nucleotides and the exclusion of this exon shifts the encoded mouse TMEM16A out of frame at amino acid I563 [GenBank: NP_848757], truncating the protein from 956 to 596 amino acids. Exon 18 encodes the fifth transmembrane spanning domain of TMEM16A [35] and exclusion of exon 18 would result in a C-terminus truncated TMEM16A protein containing four membrane spanning domains (Figure 5A). Translation of mouse TMEM16A yields a protein of ~110kDa. The exclusion of exon 18 in the mouse transcript could result in a truncated TMEM16A protein of ~60 -75 kDa (depending upon which other alternative exons reported in this study are also included or excluded). Interestingly, two independent studies have reported an additional signal on Western blots for TMEM16A [25,36] which were much lower (~80 kDa) than the expected 110 kDa. From the results presented in this study, it is possible that the lower molecular weight band corresponds to an alternatively spliced transcript that encodes a truncated TMEM16A protein.

The Δ3'exon 1 and Δ3'exon 21 alternative donor sites are the result of wobble splicing occurring at donor splice sites located in close proximity, commonly known as tandem splice sites. Alternative splicing at tandem splice sites occurs frequently in many species and can result in subtle variations in transcripts and the proteins that they encode [37]. Use of the *Tmem16a *Δ3'exon 1 splice site, results in the exclusion of the last 3 nucleotides (GTG) of exon 1. The mouse *Tmem16a *genomic sequence was queried against the TassDB database of alternative tandem splice sites [38], and the Δ3'exon 1 was recognized as tandem splicing at donor splice site with the motif GYNGYN. Indeed, a BLASTn search of the EST database revealed 27 ESTs without this 3' GTG and two ESTs that contained it. This alternative splice event is not expected to alter TMEM16A protein since exon 1 is non-coding. Skipping of exon 1 and part of exon 2 has been reported in human TMEM16A [13], however, Ferrera et al failed to identify transcripts lacking this region [22] and considered that its splicing may be under the control of an alternative promoter and influenced by certain physiological or pathophysiological conditions. Indeed, a recent study showed that a novel TMEM16A variant with an alternative 5' end is highly expressed in gastric smooth muscle of patients with diabetic gastroparesis compared with controls [23]. Apart from Δ3'exon 1 tandem splice site, our study did not identify additional variation at the 5'end. The Δ3'exon 21 tandem splice site would result in 4 additional (GTGA) nucleotides at the 3' end of exon 21. This is predicted to shift the protein out of frame at amino acid M702 [GenBank: NP_848757], resulting in a truncated TMEM16A protein with an altered C-terminus of ~84 kDa. According to TassDB2 [39] this tandem splice site is conserved in human, dog and chicken *Tmem16a *sequence.

### Distribution of novel *Tmem16a *alternative splice variants in other tissues

This study focused on identifying *Tmem16a *splice variants in heart. We extended this analysis to investigate if these novel variants are expressed in other mouse tissues from which a calcium-activated chloride current can be recorded. Initially, we confirmed *Tmem16a *expression in mouse brain, lung, stomach (antrum), small intestine, colon, kidney and bladder tissues (data not shown). All organs were dissected out with their attached vasculature. Total RNA obtained from each of these organs therefore contains a mixed population of cells. We then examined the expression of the following exons: +6b, -10, +13, +13b, -14, +15 and -18. The distribution of *Tmem16a *alternative exon variants in these mouse tissues is summarized (Table 4). In addition to heart, all of the tissues examined express the following variants; +6b, -10, +13, +13b and +15. The -14 exon variant was only detected in heart, lung, small intestine and colon. The -18 exon variant was detected in all tissues examined apart from kidney. The molecular diversity of *Tmem16a *may contribute to numerous proteomic variations of TMEM16A channels that may fine-tune channel functions in specific tissue types.

**Table 4:** Distribution of *Tmem16a *exon variants in mouse tissues

	heart	brain	lung	stomach	s.int.	colon	kidney	bladder
**+ exon 6b**	++	+++	+++	+++	+++	+++	+++	+
**- exon 10**	+	+	+	+	+	+	+	+
**+ exon 13**	+++	+++	+++	+++	+++	+++	+++	++
**+ exon 13b**	++	+++	+++	++	+++	+++	++	+
**- exon 14**	+	n.d.	+	n.d.	+	+	n.d.	n.d.
**+ exon 15**	++	++	+	+++	+++	+++	+++	+
**- exon 18**	+	+	+	+	+++	+++	n.d.	+

s.int. (small intestine) +++ indicates high, ++ moderate and + low expression. n.d., not detected.

## Conclusion

This report provides details of *Tmem16a *genomic organization and our analyses have detected previously unidentified alternative exons. We identified *Tmem16a *transcript variants consisting of different exon usage events involving exons 6b, 10, 13, 14, 15 and 18. Many of these exons are expressed in various combinations. A number of other splicing events including a novel exon 13b, tandem splice sites of exon 1 and 21 and two intron retention events were also identified. The alternative splicing events indentified here indicate that a varied population of TMEM16A channels is expressed in mouse heart and other tissues. The heart is a highly heterogeneous tissue composed of numerous cell types tailored for specialized function. It is possible that many *Tmem16a *variants can be co-expressed in the same cells, or differentially distributed in distinct cell types within the myocardium. Determining the localization of different *Tmem16a *isoforms within tissues has important implications with regard to the function of *Tmem16a*. The functional consequence of TMEM16A channel diversity remains to be determined. We can speculate however, that TMEM16A variants would create channels with altered biophysical properties, such as that reported for exon 6b, 13 and 15 in TMEM16A [22,23]. It is also possible that regulation of the channels may be altered since several of these alternatively spliced exons contain phosphorylation sites and interacting domains, for example, PKC and CamKII (exon 18), PKA (exon 6b), Erk-D domain (exon 10) and a Src-SH3 domain (exon 15). TMEM16A channels have been shown to form homodimers [40,41], therefore, it is likely that TMEM16A variants may interact with each other to form functional channels in a similar manner to that reported for the hERG family [42]. Another possibility is that alternatively spliced isoforms may associate with additional accessory proteins. It is evident from this study that alternative splicing is an exquisite mechanism that operates to diversify TMEM16A channel structures by the combinatorial selection of alternatively spliced events. Future recombinant expression of different *Tmem16a *variants will reveal their physiological role.

## Authors' contributions

FCB and KEOD conceived the design of the study and prepared the manuscript. KEOD and RAP isolated RNA, constructed the libraries and prepared samples for sequencing. KEOD analyzed the sequencing data. All authors have read and approved the final manuscript.

## Supplementary Material

Additional file 1 **Tables S1 and S2**. **TABLE S1: Primers used for RT-PCR of Tmem16 paralogs**. This table lists the GenBank accession number for *Gapdh *and *Tmem16 *paralogs, the oligonucleotide sequence of each primer and the expected amplicon sizes. **TABLE S2: Primers used for RT-PCR of Tmem16a exon variants**. This table lists the primer name, the oligonucleotide sequence of each primer, the annealing temperature of each primer set and the expected amplicon sizes. Click here for file

## References

[B1] Black DL Mechanisms of alternative pre-messenger RNA splicing Annu Rev Biochem 2003 72 291 336 10.1146/annurev.biochem.72.121801.161720 12626338

[B2] Britton FC Wang GL Huang ZM Ye L Horowitz B Hume JR Duan D Functional characterization of novel alternatively spliced ClC-2 chloride channel variants in the heart J Biol Chem 2005 280 25871 25880 10.1074/jbc.M502826200 15883157

[B3] Cunha SR Le SS Schott JJ Mohler PJ Exon organization and novel alternative splicing of the human ANK2 gene: implications for cardiac function and human cardiac disease J Mol Cell Cardiol 2008 45 6 724 734 10.1016/j.yjmcc.2008.08.005 18790697 PMC2630508

[B4] Dally S Corvazier E Bredoux R Bobe R Enouf J Multiple and diverse coexpression, location, and regulation of additional SERCA2 and SERCA3 isoforms in nonfailing and failing human heart J Mol Cell Cardiol 2010 48 4 633 644 10.1016/j.yjmcc.2009.11.012 19962989

[B5] George CH Rogers SA Bertrand BM Tunwell RE Thomas NL Steele DS Cox EV Pepper C Hazeel CJ Claycomb WC Lai FA Alternative splicing of ryanodine receptors modulates cardiomyocyte Ca2+ signaling and susceptibility to apoptosis Circ Res 2007 100 6 874 883 10.1161/01.RES.0000260804.77807.cf 17322175

[B6] Kupershmidt S Snyders DJ Raes A Roden DM A K+ channel splice variant common in human heart lacks a C-terminal domain required for expression of rapidly activating delayed rectifier current J Biol Chem 1998 273 42 27231 27235 10.1074/jbc.273.42.27231 9765245

[B7] Liao P Yong TF Liang MC Yue DT Soong TW Splicing for alternative structures of Cav1.2 Ca2+ channels in cardiac and smooth muscles Cardiovasc Res 2005 68 2 197 203 10.1016/j.cardiores.2005.06.024 16051206

[B8] Schroeter A Walzik S Blechschmidt S Haufe V Benndorf K Zimmer T Structure and function of splice variants of the cardiac voltage-gated sodium channel Na(v)1.5 J Mol Cell Cardiol 2010 49 1 16 24 10.1016/j.yjmcc.2010.04.004 20398673

[B9] Xu Y Dong PH Zhang Z Ahmmed GU Chiamvimonvat N Presence of a calcium-activated chloride current in mouse ventricular myocytes Am J Physiol Heart Circ Physiol 2002 283 H302 H314 12063303 10.1152/ajpheart.00044.2002

[B10] Hiraoka M Kawano S Hirano Y Furukawa T Role of cardiac chloride currents in changes in action potential characteristics and arrhythmias Cardiovasc Res 1998 40 23 33 10.1016/S0008-6363(98)00173-4 9876314

[B11] Hume JR Duan D Collier ML Yamazaki J Horowitz B Anion transport in heart Physiol Rev 2000 80 31 81 10617765 10.1152/physrev.2000.80.1.31

[B12] Sorota S Insights into the structure, distribution and function of the cardiac chloride channels Cardiovasc Res 1999 42 361 376 10.1016/S0008-6363(99)00039-5 10533573

[B13] Caputo A Caci E Ferrera L Pedemonte N Barsanti C Sondo E Pfeffer U Ravazzolo R Zegarra-Moran O Galietta LJ TMEM16A, a membrane protein associated with calcium-dependent chloride channel activity Science 2008 322 5901 590 594 10.1126/science.1163518 18772398

[B14] Schroeder BC Cheng T Jan YN Jan LY Expression cloning of TMEM16A as a calcium-activated chloride channel subunit Cell 2008 134 1019 1029 10.1016/j.cell.2008.09.003 18805094 PMC2651354

[B15] Yang YD Cho H Koo JY Tak MH Cho Y Shim WS Park SP Lee J Lee B Kim BM Raouf R Shin YK Oh U TMEM16A confers receptor-activated calcium-dependent chloride conductance Nature 2008 455 7217 1210 1215 10.1038/nature07313 18724360

[B16] Pifferi S Dibattista M Menini A TMEM16B induces chloride currents activated by calcium in mammalian cells Pflugers Arch 2009 458 6 1023 1038 10.1007/s00424-009-0684-9 19475416

[B17] Schreiber R Uliyakina I Kongsuphol P Warth R Mirza M Martins JR Kunzelmann K Expression and function of epithelial anoctamins J Biol Chem 2010 285 10 7838 7845 10.1074/jbc.M109.065367 20056604 PMC2844227

[B18] Stohr H Heisig JB Benz PM Schoberl S Milenkovic VM Strauss O Aartsen WM Wijnholds J Weber BH Schulz HL TMEM16B, a novel protein with calcium-dependent chloride channel activity, associates with a presynaptic protein complex in photoreceptor terminals J Neurosci 2009 29 6809 6818 10.1523/JNEUROSCI.5546-08.2009 19474308 PMC6665584

[B19] Hartzell C Putzier I Arreola J Calcium-activated chloride channels Annu Rev Physiol 2005 67 719 758 10.1146/annurev.physiol.67.032003.154341 15709976

[B20] Huang X Godfrey TE Gooding WE McCarty KS Jr Gollin SM Comprehensive genome and transcriptome analysis of the 11q13 amplicon in human oral cancer and synteny to the 7F5 amplicon in murine oral carcinoma Genes Chromosomes Cancer 2006 45 1058 1069 10.1002/gcc.20371 16906560

[B21] O'Driscoll KE Hatton WJ Britton FC Alternative splicing of the murine Tmem16a transcript in heart FASEB J 2010 24 28

[B22] Ferrera L Caputo A Ubby I Bussani E Zegarra-Moran O Ravazzolo R Pagani F Galietta LJ Regulation of TMEM16A chloride channel properties by alternative splicing J Biol Chem 2009 284 48 33360 33368 10.1074/jbc.M109.046607 19819874 PMC2785179

[B23] Mazzone A Bernard CE Strege PR Beyder A Galietta LJ Pasricha PJ Rae JL Parkman HP Linden DR Szurszewski JH Ordog T Gibbons SJ Farrugia G Altered expression of Ano1 variants in human diabetic gastroparesis J Biol Chem 2011 286 15 13393 13403 10.1074/jbc.M110.196089 21349842 PMC3075685

[B24] Hwang SJ Blair PJ Britton FC O'Driscoll KE Hennig G Bayguinov YR Rock JR Harfe BD Sanders KM Ward SM Expression of anoctamin 1/TMEM16A by interstitial cells of Cajal is fundamental for slow wave activity in gastrointestinal muscles J Physiol 2009 587 4887 4904 10.1113/jphysiol.2009.176198 19687122 PMC2770154

[B25] Davis AJ Forrest AS Jepps TA Valencik ML Wiwchar M Singer CA Sones WR Greenwood IA Leblanc N Expression profile and protein translation of TMEM16A in murine smooth muscle Am J Physiol Cell Physiol 2010 299 5 C948 C959 10.1152/ajpcell.00018.2010 20686072 PMC2980309

[B26] Kozak M Interpreting cDNA sequences: some insights from studies on translation Mamm Genome 1996 7 563 574 10.1007/s003359900171 8679005

[B27] Smith CW Valcarcel J Alternative pre-mRNA splicing: the logic of combinatorial control Trends Biochem Sci 2000 25 8 381 388 10.1016/S0968-0004(00)01604-2 10916158

[B28] Reed R Maniatis T Intron sequences involved in lariat formation during pre-mRNA splicing Cell 1985 41 1 95 105 10.1016/0092-8674(85)90064-9 3888410

[B29] Rautmann G Breathnach R A role for branchpoints in splicing in vivo Nature 1985 315 6018 430 432 10.1038/315430a0 4000270

[B30] Coolidge CJ Seely RJ Patton JG Functional analysis of the polypyrimidine tract in pre-mRNA splicing Nucleic Acids Res 1997 25 4 888 896 10.1093/nar/25.4.888 9016643 PMC146492

[B31] Sakabe NJ de Souza SJ Sequence features responsible for intron retention in human BMC Genomics 2007 8 59 10.1186/1471-2164-8-59 17324281 PMC1831480

[B32] Lareau LF Green RE Bhatnagar RS Brenner SE The evolving roles of alternative splicing Curr Opin Struct Biol 2004 14 3 273 282 10.1016/j.sbi.2004.05.002 15193306

[B33] Bell TJ Miyashiro KY Sul JY McCullough R Buckley PT Jochems J Meaney DF Haydon P Cantor C Parsons TD Eberwine J Cytoplasmic BK(Ca) channel intron-containing mRNAs contribute to the intrinsic excitability of hippocampal neurons Proc Natl Acad Sci USA 2008 105 6 1901 1906 10.1073/pnas.0711796105 18250327 PMC2538856

[B34] Bell TJ Miyashiro KY Sul JY Buckley PT Lee MT McCullough R Jochems J Kim J Cantor CR Parsons TD Eberwine JH Intron retention facilitates splice variant diversity in calcium-activated big potassium channel populations Proc Natl Acad Sci USA 2010 107 49 21152 21157 10.1073/pnas.1015264107 21078998 PMC3000244

[B35] Hartzell HC Yu K Xiao Q Chien LT Qu Z Anoctamin/TMEM16 family members are Ca2+-activated Cl- channels J Physiol 2009 587 2127 2139 10.1113/jphysiol.2008.163709 19015192 PMC2697287

[B36] He Q Halm ST Zhang J Halm DR Activation of the basolateral membrane Cl- conductance essential for electrogenic K+ secretion suppresses electrogenic Cl- secretion Exp Physiol 2011 96 3 305 316 21169331 10.1113/expphysiol.2010.055038 PMC3041851

[B37] Hiller M Platzer M Widespread and subtle: alternative splicing at short-distance tandem sites Trends Genet 2008 24 5 246 255 10.1016/j.tig.2008.03.003 18394746

[B38] Hiller M Nikolajewa S Huse K Szafranski K Rosenstiel P Schuster S Backofen R Platzer M TassDB: a database of alternative tandem splice sites Nucleic Acids Res 2007 35 Database D188 D192 10.1093/nar/gkl762 PMC1669710 17142241

[B39] Sinha R Lenser T Jahn N Gausmann U Friedel S Szafranski K Huse K Rosenstiel P Hampe J Schuster S Hiller M Backofen R Platzer M TassDB2 - A comprehensive database of subtle alternative splicing events BMC Bioinformatics 2010 11 216 10.1186/1471-2105-11-216 20429909 PMC2878309

[B40] Fallah G Romer T Detro-Dassen S Braam U Markwardt F Schmalzing G TMEM16A(a)/anoctamin-1 shares a homodimeric architecture with CLC chloride channels Mol Cell Proteomics 2011 10 2 M110 20974900 10.1074/mcp.M110.004697 PMC3033684

[B41] Sheridan JT Worthington EN Yu K Gabriel SE Hartzell HC Tarran R Characterization of the Oligomeric Structure of the Ca2+-activated Cl- Channel Ano1/TMEM16A J Biol Chem 2011 286 2 1381 1388 10.1074/jbc.M110.174847 21056985 PMC3020746

[B42] Guasti L Crociani O Redaelli E Pillozzi S Polvani S Masselli M Mello T Galli A Amedei A Wymore RS Wanke E Arcangeli A Identification of a posttranslational mechanism for the regulation of hERG1 K+ channel expression and hERG1 current density in tumor cells Mol Cell Biol 2008 28 16 5043 5060 10.1128/MCB.00304-08 18559421 PMC2519704

