# Supplementary material for: Increased complexity of Tmem16a/Anoctamin 1 transcript alternative splicing
Source: BMC Mol Biol. 2011 Aug 8;12:35. doi: 10.1186/1471-2199-12-35 (PMC3170211; doi:10.1186/1471-2199-12-35)
Supplement: Additional file 1 — Tables S1 and S2. TABLE S1: Primers used for RT-PCR of Tmem16 paralogs. This table lists the GenBank accession number for Gapdh and Tmem16 paralogs, the oligonucleotide sequence of each primer and the expected amplicon sizes. TABLE S2: Primers used for RT-PCR of Tmem16a exon variants. This table lists the primer name, the oligonucleotide sequence of each primer, the annealing temperature of each primer set and the expected amplicon sizes. [file 1471-2199-12-35-S1.DOC]

**TABLE S1: Primers used for RT-PCR of Tmem16 paralogs**

**Gene Accession no. Oligonucleotide sequences (5’-3’) Amplicon size**

| *Gapdh* | NM_008084 | GTCTTCACCACCATGGAGA (sense)  AAGCAGTTGGTGGTGCAG (antisense) | 170 bp |
| --- | --- | --- | --- |
| *Tmem16a* | NM_178642 | TCGGCCCGGTGACTACGTGTACATC (sense)  GGCTCTGTCTGCGCAGCTTCAGGTA (antisense) | 196 bp |
| *Tmem16b* | NM_153589 | GCCTTCACCATGTGTCCCCTGTGTG (sense)  GCCCAACCGCATCTGGAGTCTCTTC (antisense) | 183 bp |
| *Tmem16c* | NM_001081556 | GCCTTCCGATCAAAACCCATGGACC (sense)  CCGACAACTGCAGCAGGAATCAGCA (antisense) | 190 bp |
| *Tmem16d* | NM_178773 | CCCATGGAGCTGTAAACCACCGACA (sense)  AATGAAGGCTGCGGGGAAGAGCA (antisense) | 173 bp |
| *Tmem16e* | NM_177694 | TGGAGCCACACATGCCTCTATGCCA (sense)  CAGGGTGGCTTCGCTTTCCATGAAG (antisense) | 167 bp |
| *Tmem16f* | NM_175344 | TGCTTGGCTGGGCTATTACACGCAG (sense)  CCTCCAGAACGGACACAGCCTGTCA (antisense) | 181 bp |
| *Tmem16g* | NM_207031 | TGAATTTCTACGCCTCGCCCGTGTA (sense)  TCTGCTTGCCCACCATGATGACCA (antisense) | 167 bp |
| *Tmem16h* | XM_889480 | CGACGCGGCTGGAAGACAGAAAAG (sense)  GCCAGTGGGAAGGCAGAGGAGAAGA (antisense) | 195 bp |
| *Tmem16j* | NM_178381 | CTGTTGGCGCTCTTCAGCAACCTTG (sense)  GGCAATGACTGCCAGCACTCCAATG (antisense) | 150 bp |
| *Tmem16k* | NM_133979 | TCTGGCATCGTGACCCAAGTGTTCC (sense)  TGATGGCCATGGGGATTAAGGCAA (antisense) | 193 bp |

*Gapdh* (glyceraldehyde-3-phosphate dehydrogenase). *Tmem16a*-*k* correspond to *mus musculus* *Tmem16* paralogs.

**TABLE S2: Primers used for RT-PCR of Tmem16 exon variants**

**Primer Oligonucleotide sequences (5’-3’) Ta Amplicon size**

| ± exon 6b | AGGCCTCCTGAAAACCATCAACTCG (sense)  GACTCCGTAACTTGCCCATTCCTCA (antisense) | 59 | 367 or 433 bp |
| --- | --- | --- | --- |
| ± exon 13 | GCCACTTTCATGGAGCACTG (sense)  CACCTTGTCGGTCTCTTTGT (antisense) | 55 | 165 or 177 bp |
| ± exon 15 | ATGAAGCCAGAGTCTTAGAGAAGT (sense)  AAACTTCATCCAGCAGAATGAT (antisense) | 55 | 297 or 375 bp |
| + exon 6b | GCTGAGCACAGGCCACAGACCACAA (sense)  CCTTCTTCCCTCTCCTTGCCCCATG (antisense) | 59 | 192 bp |
| - exon 6b | CAGCATGGGTATCACCAGCCTCCTG (sense)  TTCCTGACCAGGTCAATGGGCTGG (antisense) | 59 | 174 bp |
| + exon 13 | GATGAGCTCAGCCTGTGCCACAG (sense)  TGACAGCTTCCTCCTCCTCCTCG (antisense) | 59 | 182 bp |
| - exon 13 | CCCTGCCACCGTCTTCTTCTCTGTG (sense)  TGCTCTGGGATGATCCTCCTCCTCC (antisense) | 59 | 139 bp |
| + exon 15 | GACAGCCATGGCAGGGGTGAAATTG (sense)  AGACGGGGAGGAGTTCATGGCCAAG (antisense) | 59 | 187 bp |
| - exon 15 | AGAGACCGACAAGGTGAAGCTGACC (sense)  CGTGACTGTAACCCGGATGTTGGA (antisense) | 59 | 196 bp |
| - exon 10 | TGACCTGGTCAGTATGGAGATG (sense)  ATCTGCTTCCGTTTCCAGTG (antisense) | 54 | 210 bp |
| +5’E13b  +3’E13b  -exon 14  -exon 18 | CATGTGTCCTCTGTGTGACAAGA (sense)  GATGTAGCAAAAGGCAGCAAG (antisense)  AGGAGCACCTTCTGCTGAATTCC (sense)  AGGTCAGCTTCACCTTGTCGGTC (antisense)  CTGCAGCTACTGGAAGATGAGC (sense)  ACCTTGTCGGTCTTGACAGCT (antisense)  TCATGATCGCAGTGACATTTGC (sense)  GACCAACAAACCAATCTTGGTGA (antisense) | 57  57  57  55 | 290 bp  219 bp  210 bp  218 bp |
